# Fabrication of Electrospun Double Layered Biomimetic Collagen–Chitosan Polymeric Membranes with Zinc-Doped Mesoporous Bioactive Glass Additives

**DOI:** 10.3390/polym16142066

**Published:** 2024-07-19

**Authors:** Dilan Altan, Ali Can Özarslan, Cem Özel, Kadriye Tuzlakoğlu, Yesim Muge Sahin, Sevil Yücel

**Affiliations:** 1 Faculty of Chemical and Metallurgical Engineering, Department of Bioengineering, Yildiz Technical University, 34220 Istanbul, Türkiye; alicanozarslan@gmail.com (A.C.Ö.); cemozel@yildiz.edu.tr (C.Ö.); yuce.sevil@gmail.com (S.Y.); 2 Health Biotechnology Joint Research and Application Center of Excellence, 34903 Istanbul, Türkiye; 3 Department of Polymer Engineering, Yalova University, 77200 Yalova, Türkiye; ktuzlakoglu@yalova.edu.tr; 4 Polymer Technologies and Composite Application and Research Center, Istanbul Arel University, 34537 Istanbul, Türkiye; ymugesahin@arel.edu.tr; 5 Faculty of Engineering, Department of Biomedical Engineering, Istanbul Arel University, 34537 Istanbul, Türkiye

**Keywords:** collagen, chitosan, bioactive glass, electrospinning, guided bone regeneration, zinc

## Abstract

Several therapeutic approaches have been developed to promote bone regeneration, including guided bone regeneration (GBR), where barrier membranes play a crucial role in segregating soft tissue and facilitating bone growth. This study emphasizes the importance of considering specific tissue requirements in the design of materials for tissue regeneration, with a focus on the development of a double-layered membrane to mimic both soft and hard tissues within the context of GBR. The hard tissue-facing layer comprises collagen and zinc-doped bioactive glass to support bone tissue regeneration, while the soft tissue-facing layer combines collagen and chitosan. The electrospinning technique was employed to achieve the production of nanofibers resembling extracellular matrix fibers. The production of nano-sized (~116 nm) bioactive glasses was achieved by microemulsion assisted sol-gel method. The bioactive glass-containing layers developed hydroxyapatite on their surfaces starting from the first week of simulated body fluid (SBF) immersion, demonstrating that the membranes possessed favorable bioactivity properties. Moreover, all membranes exhibited distinct degradation behaviors in various mediums. However, weight loss exceeding 50% was observed in all tested samples after four weeks in both SBF and phosphate-buffered saline (PBS). The double-layered membranes were also subjected to mechanical testing, revealing a tensile strength of approximately 4 MPa. The double-layered membranes containing zinc-doped bioactive glass demonstrated cell viability of over 70% across all tested concentrations (0.2, 0.1, and 0.02 g/mL), confirming the excellent biocompatibility of the membranes. The fabricated polymer bioactive glass composite double-layered membranes are strong candidates with the potential to be utilized in tissue engineering applications.

## 1. Introduction

Guided bone regeneration (GBR) has become a standard in clinics for treating jawbone defects by employing membranes to prevent soft tissue from infiltrating bone defects [1]. This technique facilitates bone tissue growth, particularly in cases where there is not sufficient volume for dental implants, by creating an isolated area for bone regeneration. Gore-Tex, a non-degradable material based on expanded polytetrafluoroethylene (e-PTFE) fluoropolymer, has been extensively utilized as a GBR membrane. Despite its advantageous properties such as biocompatibility, ability to create space, and stability, the need for a second surgery to remove the material after recovery has been a significant drawback associated with this class of materials. Another type of non-degradable material used as a GBR membrane is titanium mesh [2]. However, despite their superior mechanical properties like e-PTFE, titanium meshes also need to be removed after recovery. The removal procedure can increase the risk of morbidity and is burdensome for the patient [3]. Another disadvantage associated with non-degradable membranes has been reported as recurrent infections occurring during membrane use, which may adversely affect new bone formation [4].

Synthetic and natural biodegradable polymers are being increasingly used as membrane materials in clinical practice to prevent the necessity of a second surgery with non-degradable membranes. The gradual degradation of degradable membranes allows for the development of new bone in areas of defects [5]. Aliphatic esters like polylactic acid (PLA), polyglycolic acid (PGA), and polycaprolactone (PCL), as well as their numerous combinations, might be noted as synthetic biodegradable polymers used for tissue engineering purposes. Although these materials demonstrate satisfactory mechanical properties, their usage is limited by the acidic degradation products they produce [6]. These products can lead to inflammation in adjacent tissues due to the enzymatic activities involved in the process [7]. Natural biodegradable polymers like collagen [8], gelatin [9], chitosan [10], silk fibroin [11], and chitin [12] are frequently utilized in the fabrication of GBR membranes. Collagen, one of the constituents of the extracellular matrix, stands out as one of the most used natural polymers in membrane fabrication, owing to its excellent cell affinity and biocompatibility. One of the most appealing characteristics of collagen for bone tissue engineering is its structural integrity that allows the accumulation of calcium phosphate and calcium carbonate minerals [13]. Chitosan is an important naturally occurring polymer with a polysaccharide structure that is produced by deacetylating chitin. Chitosan’s hemostatic, antimicrobial, biodegradable, and biocompatible properties have led to its widespread use in various tissue engineering applications [14,15]. In addition to its aforementioned advantageous properties, chitosan’s structural similarity to glycosaminoglycan, the space filling component of the natural extracellular matrix (ECM) [16], suggests that the collagen–chitosan complex used in scaffold construction for tissue engineering is expected to mimic the constituents of the native ECM.

Introducing or enhancing bioactive properties can be achieved by incorporating bioactive compounds into the membrane structure during fabrication of membranes. Bioactive glasses are widely utilized in tissue engineering applications for their unique properties. This category of materials, also recognized as biodegradable glass materials, is extensively researched. They demonstrate bioactivity by facilitating the formation of apatite on their surface [17]. The bone-stimulating properties of bioactive glasses can be enhanced with various elemental additions, such as magnesium (Mg), zinc (Zn), strontium (Sr), and silver (Ag) [18].

In this study, zinc-doped bioactive glasses produced by the microemulsion assisted sol-gel method were added to membranes to enhance their bioactivity and stimulate bone development. Bioactive glasses synthesized through the microemulsion technique exhibit reduced susceptibility to agglomeration in contrast to other bioactive glasses, owing to their high surface areas [19,20], which is an important feature for uniform distribution within the membrane structure during production, allowing the membrane to exhibit similar properties and bioactivity throughout. Although found in small amounts in the body (75–125 µg/dL in serum), zinc performs various functions related to the immune system, cell division, fertility, and growth, and is necessary for the activity of over 300 enzymes [21]. Zinc has been proven to be an essential element for the formation, mineralization, development, and maintenance of healthy bones [22]. There are studies showing zinc’s (Zn) beneficial effects on bone formation in both in vivo and in vitro settings, and an osteoclast impairing effect of zinc has been shown [23].

Various techniques such as solvent casting/particle leaching, phase separation, and electrospinning are utilized in the production of dental membranes. However, electrospinning stands out as a simple, cost-effective, and efficient technique due to its applicability to both natural and synthetic polymers. This technique enables the production of nanofibrous structures with dimensions and high surface area resembling those of the natural ECM (5 to 500 nm) [24]. Electrospinning enables the fabrication of polymer structures with different morphologies by utilizing an electric field. During this procedure, a solution contained within a syringe is propelled along a metal needle linked to a high-voltage power source. Following solvent evaporation, the resultant nanofibers are deposited on a grounded collector surface in the form of nonwoven mats or membranes [25].

Mechanical properties and stability of nonwoven membranes can be increased with crosslinking. There several techniques such as chemical EDC (1-Ethyl-3-(3-dimethylaminopropyl)carbodiimide), NHS (N-hydroxy succinimide), glutaraldehyde (GA), biological (Genipin), physical (ultraviolet irradiation (UV), and dehydrothermal (DHT)) used for these purpose [26]. Despite concerns about the toxicity of GA, its low cost, extensive crosslinking capabilities, and short time required for crosslinking make it one of the most effective methods available [27]. The toxicity of crosslinked membranes can be reduced by using vapor crosslinking techniques, and post-treatments such as high pressure and rinsing with glycine [28].

In tissue engineering, it is crucial to utilize materials customized for the specific requirements of each tissue. Therefore, a method involving the production of layered membranes is available to address the unique needs of each tissue. This study focuses on the production of double-layered collagen-based membranes with an electrospinning technique to be used as GBR membranes. The bone-facing layer of the material is designed to mimic bone tissue by incorporating nanosized bioactive glass into the collagen fiber structure. This approach is inspired by the composite nature of bone, which consists of protein (collagen fibers) and mineral (hydroxyapatite), and the soft tissue-facing layer also aims to mimic the structure of the soft tissue, specifically targeting the protein (collagen) and glucosamine glucan (chitosan) composition by combining collagen and chitosan. In this study, the physiochemical and structural characteristics of double-layered membranes were comprehensively examined. The impact of incorporating zinc-doped bioactive glass on the bioactivity and biocompatibility behaviors of these membranes was assessed and discussed.

## 2. Materials and Methods

### 2.1. Materials

The reagents used in glass synthesis, including tetraethyl orthosilicate (TEOS), calcium nitrate tetrahydrate (Ca(NO_3_)_2_·4H_2_O), nitric acid (HNO_3_), zinc nitrate hexahydrate Zn(NO_3_)_2_·6H_2_O, hexadecyltrimethylammonium bromide (CTAB), and ethyl acetate, were obtained from Sigma Aldrich. The solvent for electrospinning, 1,1,1,3,3,3-hexafluoro-2-propanol (HFIP), was purchased from Sigma Aldrich (Steinheim, Germany); Trifluoroacetic acid (TFA) and acetic acid were obtained from Merck. Glutaraldehyde (GA) (25% in H_2_O) utilized for membrane crosslinking, glycine for removing unreacted GA residues, Dulbecco modified Eagle medium (DMEM), fetal bovine serum (FBS), phosphate buffer saline (PBS) solution, and penicillin–streptomycin used for MTT tests, were purchased from Sigma Aldrich.

### 2.2. Production of Zinc-Doped Mesoporous Bioactive Glasses

Microemulsion assisted sol-gel method was employed to synthesize mesoporous bioactive glasses nanoparticles (MBGN) based on a binary system comprising 70 mol% SiO_2_ and 30 mol% CaO. In order to incorporate additional biological functionality, Zn^2+^ ions were introduced as dopants, resulting in the synthesis of MBGN_Zn with a composition of 70 mol% SiO_2_, 25 mol% CaO, and 5 mol% ZnO, following a previously established protocol by the authors [29]. The production of nanosized bioactive glasses included dissolving hexadecyltrimethylammonium bromide (CTAB) in deionized water and adding ethyl acetate. Following the hydrolysis of TEOS, appropriate amounts of other precursors including Ca(NO_3_)_2_·4H_2_O and Zn(NO_3_)_2_·6H_2_O were introduced to the mixture. After synthesis, the resulting precipitate was washed, collected, and subjected to drying in an oven at 60 °C for 24 h. Finally, the dried materials were calcinated at 700 °C for 2 h with a heating rate of 2 °C/min.

### 2.3. Electrospinning and Crosslinking of Collagen Based Membranes 

Collagen Type I was used to prepare collagen fibers and was obtained from the tails of rats that were sacrificed after being used as a healthy control group in other studies. Briefly, rat tails were soaked in a 70% ethanol solution for 30 min to eliminate any contaminants and then immersed in a 1× PBS solution. After scraping the skin of the tails taken from the PBS solution, the tendons that became prominent and rich in collagen content were carefully separated from the tail with the help of pliers and placed in a pre-prepared 0.5 M acetic acid solution to dissolve the collagen at +4 °C. The solution was filtered with the help of multilayer gauze, dialyzed against distilled water with pH adjusted to 3 at +4 °C for 3 days, then lyophilized and stored at +4 °C [30].

To produce the soft tissue facing layer; Type 1 collagen was dissolved in HFIP with 8% (*w*/*v*) concentration. Selecting the appropriate solvent/solvent systems can be difficult, especially when working with natural polymers. Since the solubility of chitosan in HFIP was low, a solvent mixture was prepared with a volume ratio of HFIP/TFA (80:20), as suggested in the literature [31]. Simultaneously, chitosan was dissolved in the mixture. After the solutions became homogeneous, the collagen and chitosan solutions were mixed in the ratios of 90:10, 80:20, 70:30 to optimize Col-Chi ratio aiming for homogenous nanofiber production.

Bone tissue facing layer was produced as follows: Type 1 collagen was dissolved in HFIP at 8 wt.% [32]. Then, bioactive glass was added at rates of 5, 10, and 15 wt.% and optimization studies were carried out to produce membranes consisting of homogeneous and nano-sized fibers in which bioactive glasses were homogeneously distributed in the fiber structure.

Through systematic optimization of each layer, compositions were identified that exhibited the most uniform distribution of fibers and achieved the smallest fiber diameters deemed essential to produce double-layered (DL) membranes. Notably, the compositions Col-Chi (70:30) and Col-BG (90:10) were determined to be optimal for the fabrication of double-layered membranes. During the double-layered membrane fabrication process, the Col-BG layer was initially deposited onto the collector surface, followed by deposition of the Col-Chi layer atop it (Graphical Abstract).

The membranes were produced using the Fytronix ES-9000 (Elazığ, Türkiye). The flow rate during the electrospinning process was 1.0 mL/h and the distance from the needle to the aluminum foil collector was 10–15 cm and the voltage was between 15–30 kV while the rotating speed was 200–400 rpm. Relative humidity and temperature ranged from 20% to 30% and 20 to 25 °C, respectively. All electrospun nanofibrous membranes were subjected to a vacuum oven at room temperature for one week to eliminate any possible solvent residue. The membranes were crosslinked with 25% GA vapor in a desiccator. The membranes were then stored in a vacuum oven at room temperature to eliminate extra GA after being crosslinked for 24 h and subsequently rinsed with glycine to eliminate any remaining residues of GA [33].

### 2.4. Characterization

#### 2.4.1. Characterization of Bioactive Glasses and Membranes

##### Elemental Composition Analysis by X-ray Fluorescence Spectroscopy (XRF)

The elemental composition of bioactive glass nanoparticles was measured at ambient temperature by using energy dispersive X-ray fluorescence spectroscopy (EDXRF, PANalytical Minipal, Almelo, The Netherlands).

##### Surface Morphology, Particle Size, and Fiber Diameter Analysis by Scanning Electron Microscopy (SEM)

Surface morphology and particle size of the bioactive glass particles were examined by using scanning electron microscopy (SEM, Zeiss EVO LS10, Carl Zeiss, Oberkochen, Germany). SEM was also used to determine the fiber diameters and surface morphology of the electrospun membranes. The samples were sputter coated with a conductive gold layer before SEM analysis. The particle sizes of bioactive glasses were measured using ImageJ Software Version 1.8.0 (http://imagej.nih.gov/ij/; provided in the public domain by the National Institutes of Health, Bethesda, MD, USA) from 100 randomly chosen particles from different SEM images. Average fiber diameters and pore sizes were determined using ImageJ by evaluating at least 100 randomly chosen fibers/pores in SEM images. Energy dispersive X-ray spectroscopy (EDS; Carl Zeiss, SmartEDX, Oberkochen, Germany) analysis was also performed to determine the elemental composition of samples.

##### Particle Size Analysis by Dynamic Light Scattering (DLS)

The dynamic light scattering (DLS) technique (Malvern Nano ZS, Malvern Instrument Ltd., Worcestershire, UK) was used to determine the zeta potential, particle size, and size distribution of the bioactive glass particles. Particle size measuring was performed in water (0.05 mg/mL) at 25 °C and the suspensions were sonicated for 10 min before the measurements. 

##### Identification of Functional Groups by Fourier Transform Infrared Spectroscopy (FTIR)

The functional groups in the structure of both the bioactive glasses and the electrospun membranes before and after SBF exposure were identified utilizing Fourier transform infrared spectroscopy (FTIR; JASCO 6600,JASCO Ltd., Tokyo, Japan) in the wavenumber range of 2000 to 500 cm^−^^1^ and the number of scans was 15. Both the bioactive glasses and membranes were placed directly onto the attenuated total reflectance (ATR) crystal in small amounts (a few milligrams each). The crystal was cleaned thoroughly between each measurement.

##### Surface Area and Pore Characteristics Analysis by Nitrogen Adsorption-Desorption Isotherms

The assessment of the specific surface area and pore characteristics of the bioactive glasses was conducted via the analysis of N_2_-gas adsorption-desorption isotherms, employing a Brunauer–Emmett–Teller (BET) method (Micromeritics, TriStar II 3020, Norcross, GA, USA). The bioactive glass samples were weighed (0.2 g) and placed in measurement tubes and degassed under vacuum at 300 °C for 12 h prior to the adsorption experiments.

##### Crystallographic Analysis by X-ray Diffraction (XRD)

The X-ray diffraction (XRD) analyses were conducted to acquire the XRD patterns of both the bioactive glass powders and the membranes before and after simulated body fluid (SBF) exposure. These analyses were performed utilizing an XRD instrument (Rigaku, Dmax-2200, Tokyo, Japan) operating in the 2θ range of 10° to 60° and employing Cu Kα radiation. The experimental parameters included a step size of 0.010° and a dwell time of 1° per minute. 

##### Thermal Analysis by Thermogravimetric-Differential Thermal Analysis (TG-DTA)

Thermogravimetric (TG) analysis of bioactive glasses and membranes were performed with the Thermogravimetric-Differential Thermal Analyzer (TG-DTA, SIINanotechnology, SII6000 Exstar 6300, Chiba, Japan). Throughout the heating procedure, nitrogen was passed through an alumina sample container. The temperature was incrementally raised to 700 °C at a rate of 20 °C per minute. 

##### Mechanical Testing of Double-Layered Membranes

The double-layered membranes underwent mechanical testing under both dry and wet conditions at ambient settings (20 °C and 50% humidity) using tensile testing machine (Devotrans GPUG/R, İstanbul, Türkiye). The membranes were cut into 10 mm × 30 mm rectangular shapes, and measurements were conducted at a loading speed of 5 mm/min with a 20 N load cell. The tensile stress–elongation curves of the specimens were plotted using the data recorded by the machine. 

### 2.5. In Vitro Degradation of Membranes

The degradation properties of the membranes were assessed by immersing them in fresh phosphate-buffered saline (PBS) and SBF for four weeks. After being dried and weighed, the membranes (10 mm × 10 mm) were placed in falcon tubes containing 10 mL of PBS and SBF. The membranes were then rinsed with deionized water after each incubation period and dried before being weighed again. The biodegradability of the membranes was calculated using the equation [34] below (Equation (1)).

Weight loss (%) = (Wi − Wd/Wi) × 100 (1)

### 2.6. In Vitro Bioactivity of MBGN_Zn and MBGN_Zn-Incorporated Membranes

The bioactivity of bioactive glass samples was evaluated in SBF using a technique proposed for powder materials by Maçon et al. [35]. The SBF was prepared following Kokubo’s method. In brief, MBGN_Zn powders were introduced into polyethylene falcon tubes containing SBF at a concentration of 1.5 mg/mL. Subsequently, the bioactive glasses were retrieved from the SBF at the end of each time interval (ranging from 1 to 4 weeks), rinsed with distilled water and acetone to terminate any ongoing reactions, and then dried. The membrane bioactivity was assessed using the following method: the membranes were submerged in SBF, utilizing the equation formulated by Kokubo and Takedama for dense materials (Equation (2)):

Vs = Sa/10 (2)

Here, Vs represents the volume of SBF in milliliters (mL), and Sa denotes the apparent surface area of the specimen in square millimeters (mm^2^) [36].

### 2.7. In Vitro Cell Viability of Membranes

Cell viability (%) of all membranes was assessed using Saos-2 cells, employing the 3-(4,5-dimethylthiazol-2-yl)-2,5-diphenyltetrazolium (MTT) colorimetric technique following the ISO-10993-5 standard. Membrane extracts were initially prepared in cell medium at a concentration of 0.2 g/mL and then diluted to concentrations of 0.1 mg/mL and 0.2 g/mL. One milliliter of each concentration was incubated in cell medium at 37 °C for 24 h. The extracts were then introduced into 96-well plates containing Saos-2 cells (1 × 10^4^ cells per well) that had been previously incubated for 24 h at 37 °C with 5% CO_2_. After incubation of the cells with the membrane extracts, 50 μL of MTT solution was added and incubated for two hours at 37 °C with 5% CO_2_. Subsequently, the wells were rinsed three times with Dulbecco’s phosphate-buffered saline (DPBS) to remove the MTT solution. Absorbance values of each well were measured at 570 nm, and the cell viability (%) was calculated using the formula provided by the ISO 10993-5 standard procedure [37]. The averaged findings were statistically analyzed using one-way analysis of variance (ANOVA) and Tukey’s post hoc test (*p* < 0.05).

## 3. Results and Discussion

### 3.1. Characterization of MBGN_Zn

**Figure 1:**
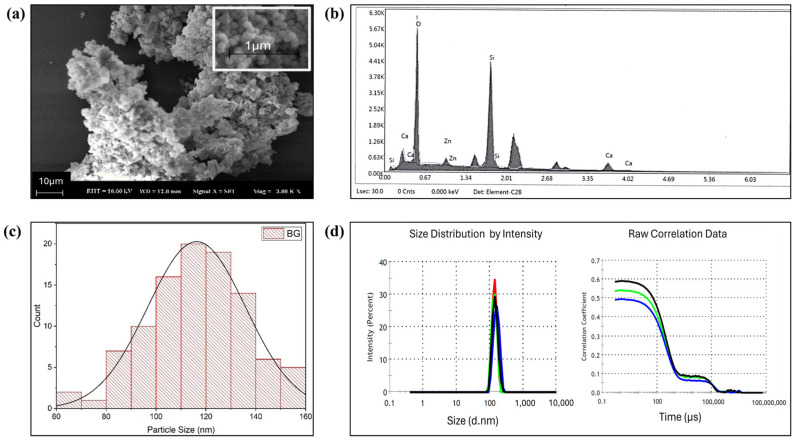
Characteristics of bioactive glass (MBGN_Zn); (**a**) SEM image, (**b**) EDS result, (**c**) particle size distribution histogram according to SEM image, (**d**) particle size distribution according to DLS analysis.

The XRF composition of the produced MBGN, expressed in oxide form, is summarized in Table 1. Molar percentages (%) of each oxide were listed based on XRF elementary analysis. The deviation of the final composition from the initially calculated values has been attributed to the washing phases of the synthesis process [38]. The presence of Si, Ca, Zn, and O elements was also confirmed with EDS analyses (Figure 1b).

As shown in Figure 1a, production of MBGN_Zn particles with spherical morphology and homogeneous size distribution was obtained via microemulsion assisted sol-gel technique. Measurements conducted using ImageJ on SEM results confirmed that the produced bioactive glass had particle sizes ranging from 60 to 160 nm, with an average particle size of 116.26 ± 19.67 nm (Figure 1c). The particle size results obtained from Zeta Sizer (Figure 1d) were slightly larger (~140 nm) than the results obtained from SEM images due to the fact that in DLS technique, the hydrodynamic diameter is measured, which measures electric double-layer and the hydration shell in addition to the particle diameter [39]. The microemulsion assisted sol-gel technique provides significant advantages over conventional sol-gel methods by producing nano-sized bioactive glasses with a narrower particle size distribution. In contrast, the surfactant-free sol-gel technique typically yields glasses of micron-sized with similar compositions [40].

**Figure 2:**
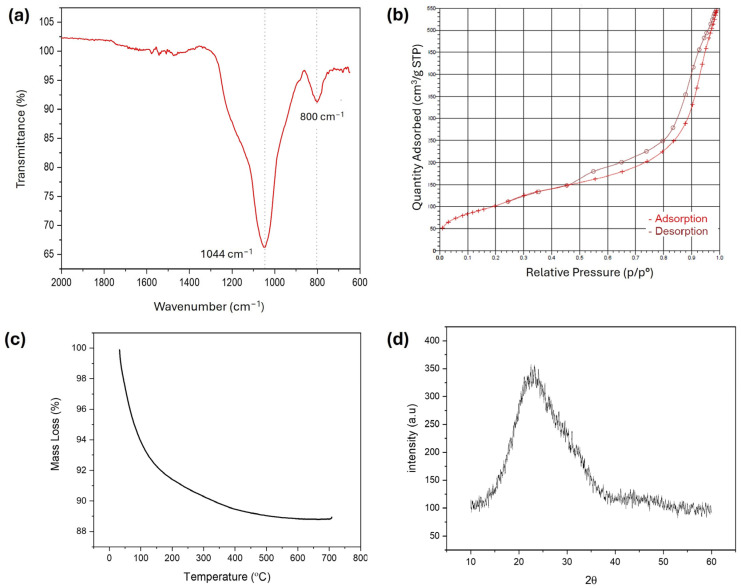
Characteristics of bioactive glass (MBGN_Zn); (**a**) FTIR spectra, (**b**) N_2_ ads.-des isotherm, (**c**) TGA curve, (**d**) XRD pattern.

The pore diameter, specific surface area and total pore volume were measured to be 5.2 nm, 368 m^2^/g, and 0.5 cm^3^/g, respectively. Besides confirming with the pore diameters between 2–50 nm, the mesoporous nature of the bioactive glasses was also indicated with the N_2_ adsorption–desorption isotherms of samples (Figure 2b). According to the International Union of Pure and Applied Chemistry (IUPAC) classification, Type IV isotherms signify mesoporous materials with non-uniform pore size distribution, displaying hysteresis loops due to capillary condensation in pores of varying sizes. FTIR spectra of bioactive glass (Figure 2a) after calcination at 700 °C for 2 h have exhibited a strong absorption peak at 1044 cm^−^^1^ which is attributed to Si–O–Si asymmetrical stretching vibrations [41]. Additionally, a Si–O–Si with bending scissoring vibrations could be observed at about 800 cm^−^^1^ which is attributed to ring structures in the glass matrix [42]. The XRD pattern of bioactive glasses is shown in Figure 2d; no diffraction peaks were observed in the graph, confirming that bioactive glasses are amorphous in form, like glass, and the broad band in the range of 15°–40° can be attributed to the amorphous silicate [29,43]. The drastic decrease in weight observed in the TGA graph of bioactive glass samples between 20–90 °C (Figure 2c) has been attributed to the removal of physically adsorbed water and residues from the bioactive glass production process [44]. Subsequently, the gradual reduction in weight observed between temperatures of 100–700 °C is attributed to the removal of internal water molecules due to the slow condensation of silanol groups [45,46].

#### Bioactivity Evaluation of MBGN_Zn

**Figure 3:**
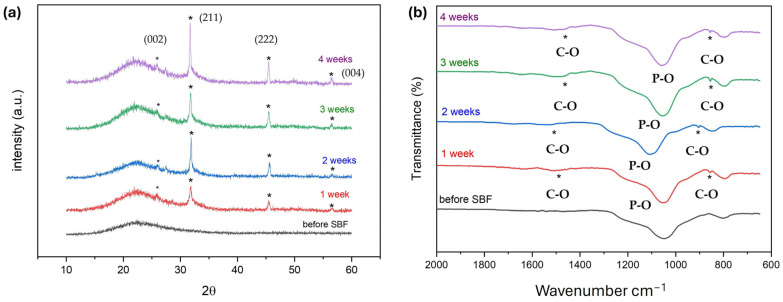
XRD patterns (**a**) and FTIR spectra (**b**) of bioactive glass (MBGN_Zn) before and after SBF incubation at the different time periods (1 week, 2 weeks, 3 weeks, 4 weeks).

Before incorporating the bioactive glasses in collagen polymer solutions to prepare the bone defect facing layer of the membranes (Col-BG), the in vitro bioactivity of glasses was examined by immersing the certain amounts of bioactive glass powders in SBF for up to 4 weeks. The assessment of the glasses’ bioactivity was conducted through XRD, FTIR analyses. The XRD patterns of the bioactive glasses soaked in the SBF solution at various times are displayed in Figure 3a. MBGN_Zn which contained 5% Zn displayed bioactivity starting from the first week. Bioactivity is defined by the development of a hydroxyapatite (HA) layer on the material’s surface, resulting from ion exchange between the material and bodily fluids. The presence of HA formation can be confirmed by the existence of specific peak characteristics of HA (Figure 3a) located at 2θ = 26, 32, 45, and 56.6° corresponding to (002), (211), (222), (004) planes of HA (JCPDS 72–1243). The intensity of these peaks was notably higher by the end of the fourth week. Previously, authors have noted the retarding effect of Zn on bioactivity, and even observed the absence of peaks associated with HA [22,47,48]. They suggested that this observation might vary depending on the process of SBF immersion. When the FTIR analyses following SBF were examined, starting from the first week, peaks corresponding to C–O bonds of carbonate groups in the HA structure were observed at wavenumbers of 1500–1400 cm^−1^ and 875–800 cm^−1^. Additionally, a shift to higher wavenumbers in the peaks occurred at wavenumbers of 1100–1000 cm^−1^ due to phosphate formation [49]. In this study we adopted an alternative method for bioactivity assessment, as recommended for powder samples. It was suggested that the bioactivities of high surface area bioactive materials produced via the sol-gel method may not align with the tests recommended by ISO for coating and disc-shaped materials [35]. Through our study, we have demonstrated that mesoporous bioactive glasses containing Zn exhibit bioactivity as early as the first week of immersion.

### 3.2. Characterization Membranes

#### 3.2.1. SEM Analyses of Electrospun Membranes 

**Figure 4:**
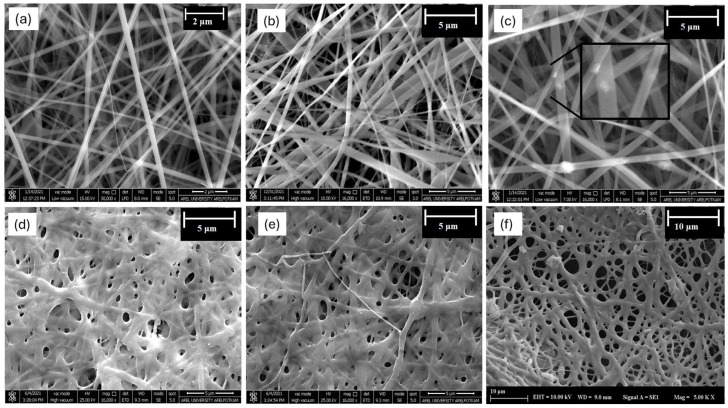
SEM images of (**a**) 100Col, (**b**) Col-Chi, (**c**) Col-BG before crosslinking, and (**d**) 100Col, (**e**) Col-Chi, (**f**) Col-BG after crosslinking.

The electrospinning technique is a method that enables the production of membranes possessing a similar pore size and fiber structure to the ECM. Research has demonstrated that the diameter of the membrane fiber can affect cellular activities such as adhesion, proliferation, and migration, attributed to a larger surface area enabling better cell-surface interaction [50]. The findings of this study revealed that the average fiber diameter of membranes was 324 ± 60 nm for 100 Col (Figure 4a), 265 ± 141 nm for 70:30 Col-Chi (Figure 4b), and 349 ± 124 nm for 90:10 Col-BG (Figure 4c) membranes. The larger fiber diameters in the membranes containing bioactive glasses have previously been attributed to the increased viscosity of the polymeric suspension due to the presence of filler particles [34]. According to Rad et al., the increase in fiber diameter observed upon the addition of bioactive glass is attributed to the agglomeration of bioactive glass particles within the fibers [51]. Conversely, the smaller diameters of chitosan-containing membranes may be linked to interactions between collagen and chitosan, rendering them miscible at a molecular level. The presence of ultra-thin fibers in the 40 nm range could result from these interactions, and another factor that has been proposed is the formation of organic salts due to the TFA solvent added to dissolve chitosan [52]. Organic salts have been reported to increase the charge density, potentially influencing the fiber diameter when added to electrospinning solutions in small amounts [53]. The fiber diameters have increased for all compositions after crosslinking. The average fiber diameter of membranes was 614 ± 251 nm for 100 Col (Figure 4d), 463 ± 175 nm for Col-Chi (Figure 4e), and 702 ± 149 nm for Col-BG (Figure 4f) membranes. The increase in fiber diameter of collagen membranes following crosslinking with GA can be ascribed to the improved structural integrity and denser arrangement of collagen fibers, resulting in the merging of fibers [54]. 

**Figure 5:**
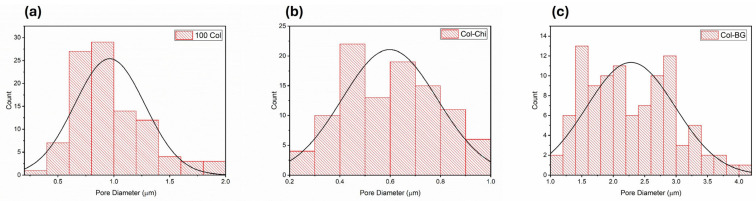
Pore size distribution histograms of (**a**) 100 Col, (**b**) Col-Chi, and (**c**) Col-BG crosslinked membranes (measurements conducted using ImageJ on SEM images).

Inhibiting the passage of microorganisms to the defect area is largely dependent on the pore size of the membrane. Randomly oriented nonwoven membranes facilitate the creation of ultrasmall pore formations [55]. It was observed that the pore sizes of the produced membranes are limited to a few microns. The pore sizes were measured as 0.972 ± 0.337 µm, 0.625 ± 0.216 µm, and 2.263 ± 0.734 µm respectively for 100 Col, Col-Chi, and Col-BG samples (Figure 5). The number and size of pores were measured to be larger for Col-BG membranes. Law et al. explained the larger pore sizes by larger fiber diameters [27]. Nelson et al. also reported a positive correlation between fiber diameter and the pore size of electrospun membranes [56].

#### 3.2.2. Bioactivity Studies of Membranes

**Figure 6:**
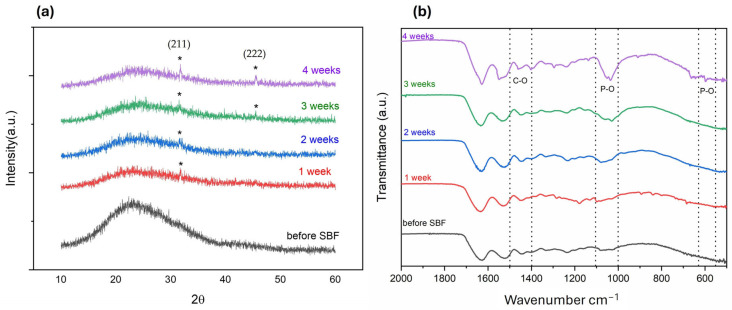
XRD patterns (* indicates HA, JCPDS; 72–1243) (**a**) and FTIR spectra (**b**) of membranes before and after SBF incubation at the different time periods (1 week, 2 weeks, 3 weeks, 4 weeks).

The bioactivity of membranes was assessed by immersing them in SBF for 4 weeks. Peaks related to the (211) plane of HA at 2θ = 32° could be observed starting from the first week of immersion, while peaks related to (222) at around 2θ = 45° were visible after 3 weeks of immersion (Figure 6a) [49,57]. Confirmation of the formation of HA on the surface of membranes during immersion in SBF was obtained from the FTIR spectra shown in Figure 6b. After 4 weeks of immersion, a peak at 600 and 570 cm^−^^1^ corresponding to the P–O bending vibrations associated with the PO_4_^3−^ group in the crystalline layer of HA was observed. Additionally, phosphate group (P–O stretching) was indicated by a peak in the region between 1100 and 1000 cm^−1^. The peaks in the region between 1500 and 1400 cm^−^^1^ were attributed to the C–O stretching, belonging to the CO_3_^2−^ groups in the carbonated HA layer [58].

**Figure 7:**
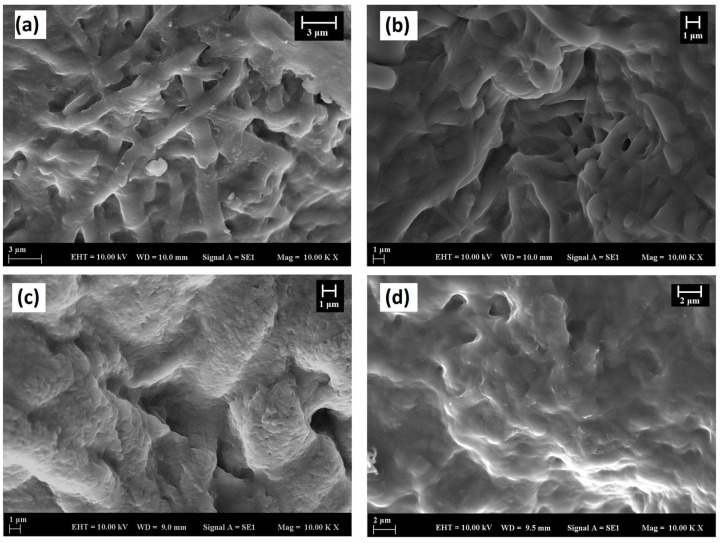
SEM images of Col-BG membranes; at the end of (**a**) 1 week, (**b**) 2 weeks, (**c**) 3 weeks, (**d**) 4 weeks in SBF.

The confirmation of HA formation was further supported by SEM analyses (Figure 7). SEM images of the membranes after immersion in SBF for 1, 2, 3, and 4 weeks showed the progression of calcium phosphate precipitates. Precipitates became visible at the end of one week, and the entire surface was covered by the end of the fourth week.

Overall, the SEM images, XRD results, and FTIR spectra provide strong evidence for the formation of HA on the membrane surfaces during immersion in SBF, confirming their bioactivity and potential for bone-bonding applications.

#### 3.2.3. Thermal Analysis of Membranes

**Figure 8:**
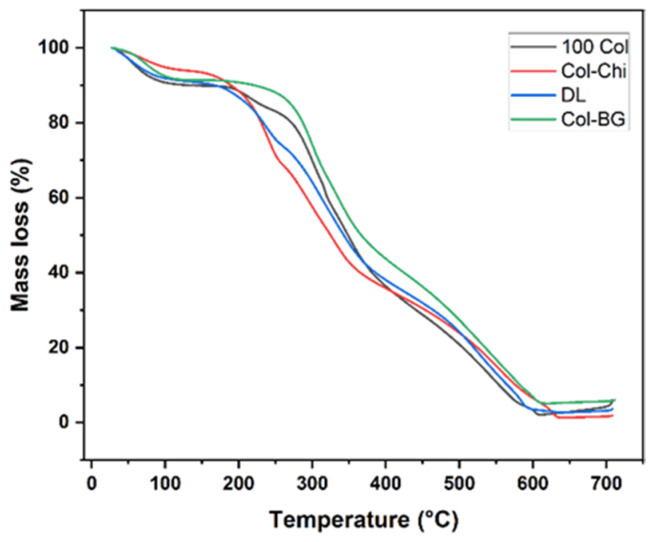
TGA curves of membranes at the constant heating rate (20 °C per minute).

The mass loss of collagen occurs in two stages: first, the desorption of physically bound water within the collagen structure, and second, the thermal degradation of polymeric chains, during which the peptide bonds within the collagen molecules begin to break down. Chitosan degrades in three stages. In addition to the two stages observed for collagen -loss of structurally bound water and thermal degradation of polymeric chains-chitosan has an extra initial step. This additional step, occurring at lower temperatures, is due to the loss of absorbed or weakly bound water and is typically recognized as the first drop on the thermogravimetric analysis (TGA) curve (Figure 8) [54].

As observed in the graph, the TGA curves of membranes containing chitosan are slightly different from those that do not contain chitosan. The observed variation has been attributed to the modified thermal stability of the composite material as a result of the interactions between hydroxyl (–OH), amino (–NH_2_), and carbonyl (–C=O) groups found in collagen and chitosan [59]. Another factor that can be implicated in the various curves is the application of acidic solvent TFA which used to increase the solubility of the chitosan [60]. It is known that the low pH causes the chitosan’s amino groups to protonate. This allows NH^3+^ in the chitosan structure and –COO^−^ in the aspartic and glutamic residues in the collagen structure to interact electrostatically, leading to a decrease in the thermal stability of Col-Chi membranes [59].

#### 3.2.4. Mechanical Properties of Membranes

**Figure 9:**
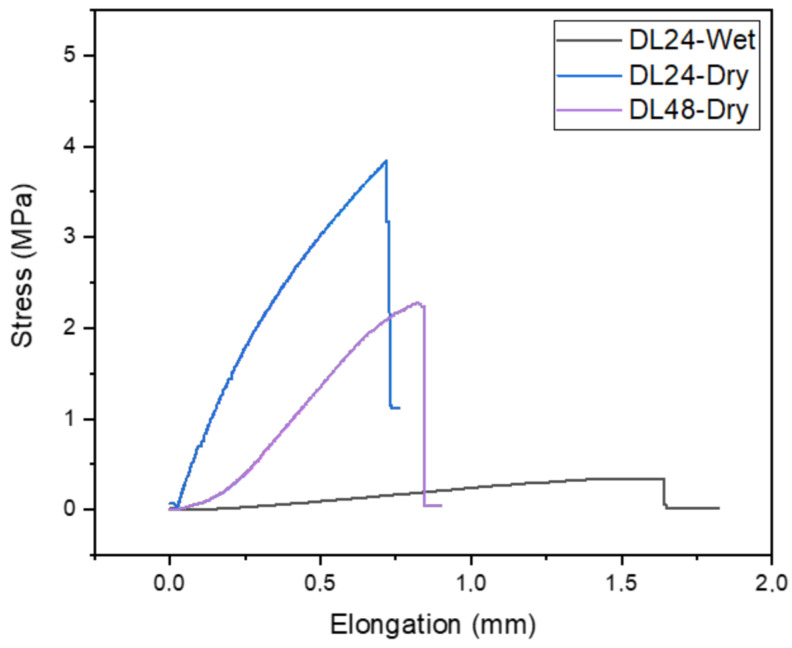
Stress-elongation graph of membranes.

Nonwoven electrospun collagen-based membranes are highly susceptible to degradation even with minimal exposure to water, and their fiber structure can be easily degraded by moisture [32]. Therefore, crosslinking of collagen-based electrospun membranes before use is important to ensure the barrier properties of the membrane and to obtain more controlled degradation behavior. Compared to synthetic polymers, collagen typically exhibits a lower tensile strength due to its natural biomaterial composition. The mechanical properties of the double-layered electrospun membranes were evaluated through tensile testing under both dry (DL24-Dry and DL48-Dry) and wet conditions (DL24-Wet). Prior to testing, the membranes were immersed in SBF for 2 min to observe their behavior under wet conditions. Additionally, double-layered membranes crosslinked for 48 h (DL48-Dry) were also tested to assess the effect of crosslinking duration on mechanical properties. Figure 9 illustrates the stress/elongation curves for the double-layered samples. The hydrated crosslinked collagen membranes displayed significantly higher elongation and lower stress compared to the dry samples. This outcome was expected, as previously mentioned in the literature, due to the weakening of non-covalent interactions by water molecules [61].

It is anticipated that the water content of the hydrated, crosslinked collagen membranes had enhance the mobility of polymer chains, leading to the observed high elongation in the hydrated samples [62]. As the crosslinking time increased, the membranes’ ability to withstand stress decreased, possibly due to the formation of a more rigid structure. It has been previously reported that an increase in crosslinking density does not always lead to an increase in mechanical properties [63]. In a study conducted by Guo et al., mechanical analysis of membranes produced via electrospinning revealed that collagen membranes had a tensile strength of 2.13 ± 0.17 MPa, which increased to 6.16 ± 1.43 MPa upon the addition of chitosan. These results highlight the significant enhancement of mechanical properties facilitated by chitosan [64].

#### 3.2.5. Biodegradation of Membranes

**Figure 10:**
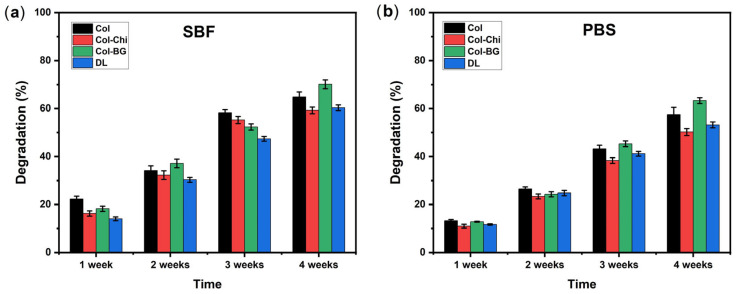
Degradation (%) of membranes over time (1, 2, 3, and 4 week(s)) in (**a**) SBF and (**b**) PBS (data were given as mean and standard deviations and indicated with error bars, *p* values < 0.05 (*n* = 3)).

The natural degradation capability of electrospun membranes is a critical property, influencing their biocompatibility and potential for tissue regeneration. Maintaining structural integrity is key for these membranes to exhibit barrier properties during the healing process [65]. When the degradation behavior of the membranes in SBF and PBS was compared, it was observed that in SBF (Figure 10a), the degradation rates for all samples (Col, Col-Chi, Col-BG, and DL) generally increased over time. Col-BG consistently exhibited the highest degradation rate among all samples, surpassing Col and Col-Chi. The increased degradation rate observed in the Col-BG samples in both PBS and SBF after a 4-week incubation period could be attributed to the presence of bioactive glasses with a high surface area in the membrane structure. This led to greater liquid adsorption, and the gradual dissolution of the glass within the membrane structure may have also contributed to the higher weight loss observed in these samples [34]. DL showed the lowest degradation rate among the samples, although it still increased steadily over the 4-week period. This could be attributed to the formation of a crosslinked layer primarily at the surface, resulting in a lower degree of crosslinking at the deeper layers of the membrane. Consequently, these deeper layers may have become more prone to degradation once the better crosslinked surface layer was removed [66]. 

In contrast, in PBS (Figure 10b), the degradation rates were generally lower compared to SBF for all samples. Col-BG still demonstrated the highest degradation rate, followed by Col-Chi, Col, and DL. However, the rate of increase in degradation over time appeared to be slower in PBS compared to SBF for all samples. Overall, the degradation behavior in SBF was more pronounced and accelerated compared to PBS, with Col-BG consistently showing the highest degradation rate across both environments. The degradation in SBF proved to be more complex, likely due to mineral accumulation on the membrane surface [67]. A well-designed GBR barrier should gradually degrade over time. Degradable membranes offer the advantage of naturally decomposing without requiring surgical removal, but there are challenges in controlling the degradation process of these collagen membranes [68].

#### 3.2.6. Cell Viability Evaluation of Membranes

**Figure 11:**
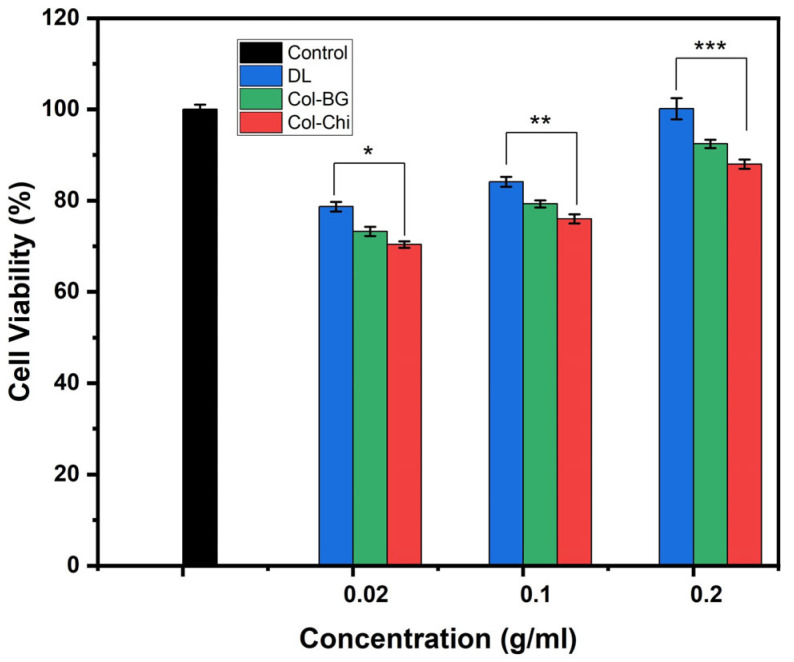
The cell viability (%) values of membranes at different extract concentrations after 24 h of incubation (data were given as mean ± standard deviations (*n* = 6)—different symbols (*, **, ***) indicated with error bars on the bar show a statistically significant difference, *p* values < 0.05).

The MTT assay is a colorimetric method commonly used to assess cell viability and proliferation. The metabolic activity of cells is measured by converting MTT, a yellow tetrazolium salt, into purple formazan crystals through mitochondrial enzyme activity. For optimal tissue regeneration, the electrospun membranes should facilitate the adhesion and proliferation of target cells. The least toxicity is a desirable property of any material in contact with the living tissues. Collagen–chitosan complexes have been demonstrated to be non-cytotoxic when tested against various cell lines using different techniques [64,69,70]. It was observed that all sample groups exhibited cell viability above 70% at various concentrations studied, indicating that the produced membranes do not have a cytotoxic effect and demonstrate biocompatible properties (Figure 11) [37]. Statistically significant differences were found between different concentrations within all sample groups (*p* < 0.05). Moreover, significant differences were noted among Col-Chi, Col-BG, and DL samples at the same concentration. Upon analyzing the results at identical concentrations, it was noted that membranes doped with bioactive glass displayed higher cell viability (%) than other samples. Particularly, samples containing double-layered membranes and bioactive glass containing membranes exhibited the highest cell viability (%). While cell viability values generally increased with concentration, this rise was more noticeable in membranes containing bioactive glass. Consequently, it can be inferred that the contribution of bioactive glass becomes more pronounced at higher concentrations. The results showed that the samples containing chitosan exhibited the lowest cell viability. Collagen has been reported to influence chitosan’s cytocompatibility, primarily by widening its pore aperture, thus enhancing chitosan’s water retention capacity. Consequently, this may facilitate cell adhesion and infiltration into the pores, promoting the formation of three-dimensional growths [69]. In a study conducted by Wang et al., it was demonstrated that a chitosan–collagen composite film enhances osteoblast functions and mineralization in MC3T3-E1 cells by increasing Erk1/2 phosphorylation, thereby enhancing Runx2 transcriptional activity. Moreover, the film induced the overexpression of osteoblastic marker genes such as Runx2 and Type I collagen in these cells [71].

## 4. Conclusions

This study highlights the significant role of tailored material design in enhancing the bone regeneration process, specifically through the application of guided bone regeneration techniques. We have developed a highly effective approach to improve tissue regeneration by designing a double-layered membrane that addresses the distinct needs of both soft and hard tissues. The bone tissue facing layer, composed of collagen and zinc-doped bioactive glass, may promote the growth of bone tissue, while the soft tissue layer, made of collagen and chitosan, may meet the needs of soft tissue. We employed electrospinning to create nanofibers that imitate the extracellular matrix, proving that the membranes displayed desirable bioactivity, biodegradability, and biocompatibility. The results indicate that the double-layered membranes made from polymer-bioactive glass composite, which were created in this research, have great potential for use in tissue engineering. These membranes may provide an advanced solution to the difficulties faced in bone regeneration in dental applications.

## Figures and Tables

**Table 1:** Theoretical and experimental molar percentages of oxides in the structure of bioactive glass (MBGN_Zn).

Oxide	Theoretical (%)	Experimental (%)
SiO_2_	70	84.62
CaO	25	11.73
ZnO	5	3.65

## Data Availability

The data presented in this study are available on request from the corresponding author.
